# A Clinicopathological Study of Surface Epithelial Tumors of the Ovary: A Retrospective Analysis at a Tertiary Care Center in Jeddah, Saudi Arabia

**DOI:** 10.7759/cureus.78200

**Published:** 2025-01-29

**Authors:** Layla S Abdullah, Badraa Alahmadi, Reema Abonab, Salma Baeisa, Bodoor Alahmadi

**Affiliations:** 1 Pathology, King Abdulaziz University, Jeddah, SAU; 2 College of Medicine, King Abdulaziz University, Jeddah, SAU; 3 Internal Medicine, King Fahad Military Medical Complex, Jeddah, SAU

**Keywords:** cancer, female, incidence, jeddah, ovarian, ovarian tumors, retrospective, saudi arabia, surface epithelial ovarian tumors, tertiary center

## Abstract

Background

Ovarian cancer is a prevalent gynecological cancer in women and one of the most common cancers globally. Factors such as the use of oral contraceptives, multiparity, and breastfeeding offer protection, while obesity, nulliparity, smoking, and genetic changes increase the risk. Ovarian cancer is asymptomatic in the early stages, typically becomes symptomatic in advanced stages, and can be divided into various histologic subtypes. This study aimed to determine the incidence of epithelial ovarian tumors according to their type and clinical presentation.

Aim

The study was conducted to determine the incidence of epithelial ovarian tumors according to their type and clinical presentation among women in a tertiary care center in Jeddah, Saudi Arabia. This is important because very few studies have been conducted in Saudi Arabia regarding the incidence of surface epithelial ovarian tumors.

Methodology

This retrospective cohort study utilized histopathological analysis to categorize ovarian neoplasms. Data on sociodemographic characteristics, histopathological findings, and clinical features were collected and analyzed from the tertiary care center to understand the distribution of surface epithelial ovarian tumors. The data were cleaned in Microsoft Excel (Microsoft Corp., Redmond, US) and analyzed using IBM SPSS Statistics version 29.0.0 (IBM Corp., Armonk, US).

Results

Our comprehensive study examined 252 women with ovarian tumors, revealing a predominant age group of 41-50 years (n=63, 25.0%), with the majority being married (n=193, 76.6%). Benign tumors were the most common (n=139, 55.2%), followed by malignant (n=87, 34.5%) and borderline (n=25, 9.9 %) tumors. The initial symptoms frequently included abdominal pain (n=105, 41.7%) and abdominal masses (n=65, 25.8%). Most tumors were primary (n=110, 43.7%). Protective factors such as breastfeeding/parity were significant (n=99, 39.3%), while nulliparity was a notable risk factor (n=31, 12.3%). The most elevated tumor marker was cancer antigen 125 (CA-125) (n=93, 36.9%). Treatment often involved total abdominal hysterectomy with bilateral salpingo-oophorectomy (TAH-BSO) (n=92, 36.5%), with some requiring additional chemotherapy (n=36, 14.3%).

Conclusion

Our study demonstrated that surface epithelial tumors, particularly serous epithelial tumors, were the most common ovarian neoplasms, with abdominal pain being a frequent initial symptom. Effective management often included surgery and chemotherapy. Breastfeeding and parity were significant protective factors and CA-125 was a prevalent tumor marker.

## Introduction

Ovarian cancer is the seventh most common malignant tumor [1], and it is one of the most prevalent gynecologic cancers, coming in third place after uterine and cervical cancers [2]. In addition, it is the fifth leading cause of cancer-related deaths among women and has a five-year survival rate of <45% [3]. The incidence varies globally according to country and ethnicity [4]. Increased risk factors have led to an upward trend in the global incidence of ovarian cancer [1]. Typically, ovarian cancer is asymptomatic in its early stages and appears at an advanced stage (stage III or IV). As symptoms of ovarian cancer are non-specific, they are easily dismissed by women at an early stage [5] as they are frequently misinterpreted as physiological changes related to pregnancy, menopause, or aging [6].

Ovarian cancer can be divided into various histological subtypes with distinct risk factors, cells of origin, molecular composition, clinical presentation, and treatment modalities. These histologic subtypes include epithelial cancers, which account for 90% of ovarian cancers, and at least five major types: high-grade serous carcinoma (HGSC) (70%), endometrioid carcinoma (EC) (10%), clear cell carcinoma (CCC) (10%), mucinous carcinoma (MC) (3%), and low-grade serous carcinoma (LGSC) (<5%) [7,8].

A retrospective study was conducted in a tertiary center in India and published in 2020, with 56 cases included, aged between 21 and 30, of which 44 were benign and 12 were malignant. Abdominal mass was the most common presentation among the patients, followed by abdominal mass and pain (27%). Regarding the types of surface epithelial tumors, the most common histopathological type of benign and malignant tumor was serous cystadenoma (50.2%), followed by serous cystadenocarcinoma (9%) [9]. In another study published in Korea, data from the Korea Central Cancer Registry, recorded between 1999 and 2012, were evaluated and published in 2016. The incidence of epithelial ovarian cancer (EOC) histological subtypes was determined. Age-standardized incidence rates (ASRs) and annual percentage changes (APCs) in incidence rates were calculated. Patient data were divided into three groups based on age (<40, 40-59, and >59 years), and the age-specific incidence rates were compared. Overall, the incidence of EOC was found increasing in Korea. Among the histologic subtypes, the incidence of CCC has increased markedly across all age groups since 1999 [10]. A further study was conducted at King Abdulaziz University Hospital (KAUH), Jeddah, Saudi Arabia, to review the clinical and pathological features of all patients who underwent staging or debulking procedures for surface EOC between October 2000 and May 2018. The total number of patients was 144, with a median age of 49 years, and the results showed that older age and papillary and clear cell carcinoma subtypes were associated with a decrease in disease-free survival (DFS) and overall survival (OS) [11].

This study aimed to determine the incidence of epithelial ovarian tumors according to their type and clinical presentation among women in a tertiary care center in Jeddah, Saudi Arabia for the reason that there have been very few studies conducted in Saudi Arabia regarding the incidence of surface epithelial ovarian tumors.

## Materials and methods

This retrospective observational study, conducted from October 20, 2023, to December 26, 2024, employed a cohort design. It aimed to assess the incidence and types of surface epithelial ovarian tumors and determine the associated risk factors, protective factors, and management of these tumors.

This study was conducted at KAUH in Jeddah, Saudi Arabia. This high-volume institution provided a robust platform for examining cases of surface epithelial ovarian tumors, ensuring consistency in patient demographics, clinical practice, and management protocols.

The study included all female patients residing in the western region of Saudi Arabia who were diagnosed with surface epithelial ovarian tumors between 2012 and 2021 at KAUH and had histopathological specimens obtained from hysterectomy with unilateral or bilateral oophorectomy, oophorectomy alone, or ovarian cystectomy, all of which were received by the histopathology department at KAUH. Eligible patients were identified through the KAUH medical records database utilizing the VIDA (VIDA Diagnostics Inc., Coralville, US) and PHOENIX (KAUH, Jeddah, Saudi Arabia) systems. Exclusion criteria included patients with other types of ovarian tumors, those diagnosed with paraovarian lesions, and those with incomplete clinical data. In total, 252 patients were included in this study.

Key variables included sociodemographic characteristics (age and marital status), pathological data (tumor type and stage), and clinical data (symptoms at presentation, tumor markers, risk factors, protective factors, and management approaches). Data extraction was performed using a standardized collection form based on information from the KAUH database.

The data were analyzed using IBM SPSS Statistics version 29.0.0 (IBM Corp., Armonk, US). A comprehensive statistical analysis encompassing both descriptive and inferential methodologies was conducted. The descriptive analysis summarized the demographic characteristics of the participants, including age, marital status, and other features of ovarian cancer.

The research protocol was approved by the KAUH Institutional Review Board. This study involved a retrospective analysis of de-identified clinical data. All data were anonymized prior to the analysis, and strict measures were taken to ensure confidentiality and data security.

## Results

Our study assessed the sociodemographic characteristics and histopathological features of 252 patients with ovarian tumors (Table 1). Notably, the age distribution revealed that the largest age group was 41-50 years (n=63, 25.0%) with a mean age of 48.2 years (SD=15.1). A significant proportion of the patients were married (n=193, 76.6%). In terms of tumor type, benign tumors were the most common (n=139, 55.2%), followed by malignant tumors (n=87, 34.5%), and borderline tumors (n=25, 9.9%). Tumor origin was predominantly primary (n=110, 43.7%), with fewer cases of secondary metastatic tumors (n=18, 7.1%)

**Table 1:** Sociodemographic characteristics of patients with ovarian tumors (n=252)

Characteristics	Variables	Number of cases (%)
Age	<20 years	8 (3.2%)
	21-30 years	25 (9.9%)
	31-40 years	45 (17.9%)
	41-50 years	63 (25.0%)
	51-60 years	52 (20.6%)
	61-70 years	38 (15.1%)
	>70 years	21 (8.3%)
	Mean (SD)	48.2 (15.1)
Marital status	Single/widow/divorced	55 (21.8%)
	Married	193 (76.6%)
Tumor type	Benign tumor	139 (55.2%)
	Borderline	25 (9.9%)
	Malignant tumor	87 (34.5%)
Tumor origin	Can't be determined/synchronous tumor	8 (3.2%)
	Primary tumor	110 (43.7%)
	Secondary tumor (metastases to tumor)	18 (7.1%)

**Figure 1:**
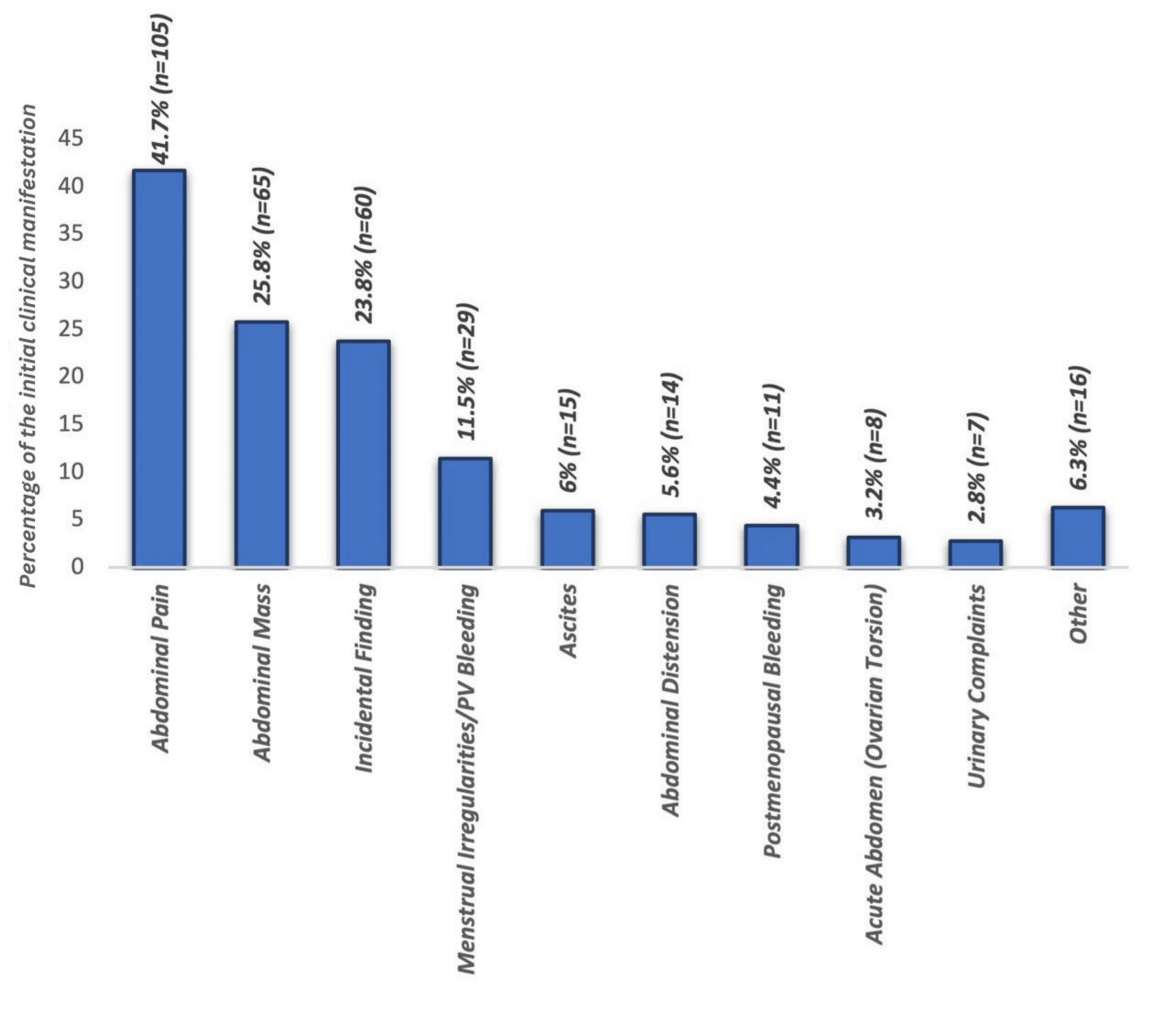
Initial clinical manifestations among patients diagnosed with ovarian tumors (n=252) 'Other' includes dyspareunia, infertility, groin pain, etc.

Figure 1 shows the initial presentation of patients with surface epithelial ovarian tumors among 252 individuals. The most common symptom reported was abdominal pain, experienced by 41.7% (n=105) of patients. This was followed by an abdominal mass noted in 25.8% (n=65) of the individuals. Other significant initial symptoms included incidental findings during other examinations (23.8%, n=60) and menstrual irregularities or heavy bleeding (11.2%, n=29). The less common symptoms included ascites (6%, n=15), abdominal distension (5.6%, n=15), and postmenopausal bleeding (4.4%, n=11). The least frequent presentations were acute abdominal pain due to ovarian torsion (3.2%, n=8), urinary complaints (2.8%, n=7), and other unspecified symptoms (6.3%, n=16). 

Table 2 provides a detailed breakdown of the histopathology of surface epithelial ovarian tumors among the patients (n=252). Notably, the serous tumors emerged as the predominant type, encompassing 154 cases (61.1%). Within this category, the most frequent subtype was serous cystadenoma, observed in 85 cases (55.2% of serous tumors), followed by HGSC in 47 cases (30.5%), serous borderline tumors in 13 cases (8.4%), and other serous tumors in nine cases (5.8%). Mucinous tumors were also significantly represented with 54 cases (21.4%), with mucinous cystadenoma being the majority (n=35, 64.8% of mucinous tumors), mucinous borderline tumors (n=9, 16.6%), mucinous adenocarcinoma (n=5, 9.2%), and other mucinous forms (n=5, 9.2%). Endometrioid tumors accounted for 30 cases (11.9%), with endometrioid adenocarcinoma not otherwise specified (NOS) being the most common (n=15, 50.0% of endometrioid tumors), followed by EC (n=8, 26.7%), endometrioid cystadenoma NOS (n=5, 16.7%), and other endometrioid forms (n=2, 6.6%). Among the less common types, seromucinous tumors included six cases (2.4%), predominantly seromucinous cystadenoma (n=5, 83.3%) and seromucinous borderline (n=1, 16.7%). Brenner tumors, all classified as Brenner tumor NOS, were present in six cases (2.4%). Clear cell tumors, the least frequent, were represented by clear cell adenocarcinoma NOS and CCC, each contributing one case (50.0% each of the clear cell categories).

**Table 2:** Histopathology of surface epithelial ovarian tumors among patients (n=252) NOS: Not otherwise specified

Nature of tumor	Type of tumor	Number of cases (%)
Type of surface epithelial ovarian tumor	Serous	154 (61.1%)
	Mucinous	54 (21.4%)
	Endometroid	30 (11.9%)
	Seromucous	6 (2.4%)
	Brenner	6 (2.4%)
	Clear cells	2 (0.8%)
Serous epithelial tumor (n=154)	Serous cystadenoma	85 (55.2%)
	High-grade serous carcinoma	47 (30.5%)
	Serous borderline tumors	13 (8.4%)
	Other	9 (5.8%)
Mucinous epithelial tumor (n=54)	Mucinous cystadenoma	35 (64.8%)
	Mucinous borderline tumor	9 (16.6%)
	Mucinous adenocarcinoma	5 (9.2%)
	Other	5 (9.2%)
Endometrioid epithelial tumor (n=30)	Endometrioid adenocarcinoma NOS	15 (50.0%)
	Endometrioid carcinoma	8 (26.7%)
	Endometrioid cystadenoma NOS	5 (16.7%)
	Others	2 (6.6%)
Clear cell epithelial tumor (n=2)	Clear cell adenocarcinoma NOS	1 (50.0%)
	Clear cell carcinoma	1 (50.0%)
Seromucinous epithelial tumor (n=6)	Seromucinous cystadenoma	5 (83.3%)
	Seromucinous borderline	1 (16.7%)
Brenner epithelial tumor (n=6)	Brenner tumor NOS	6 (100.0%)

**Figure 2:**
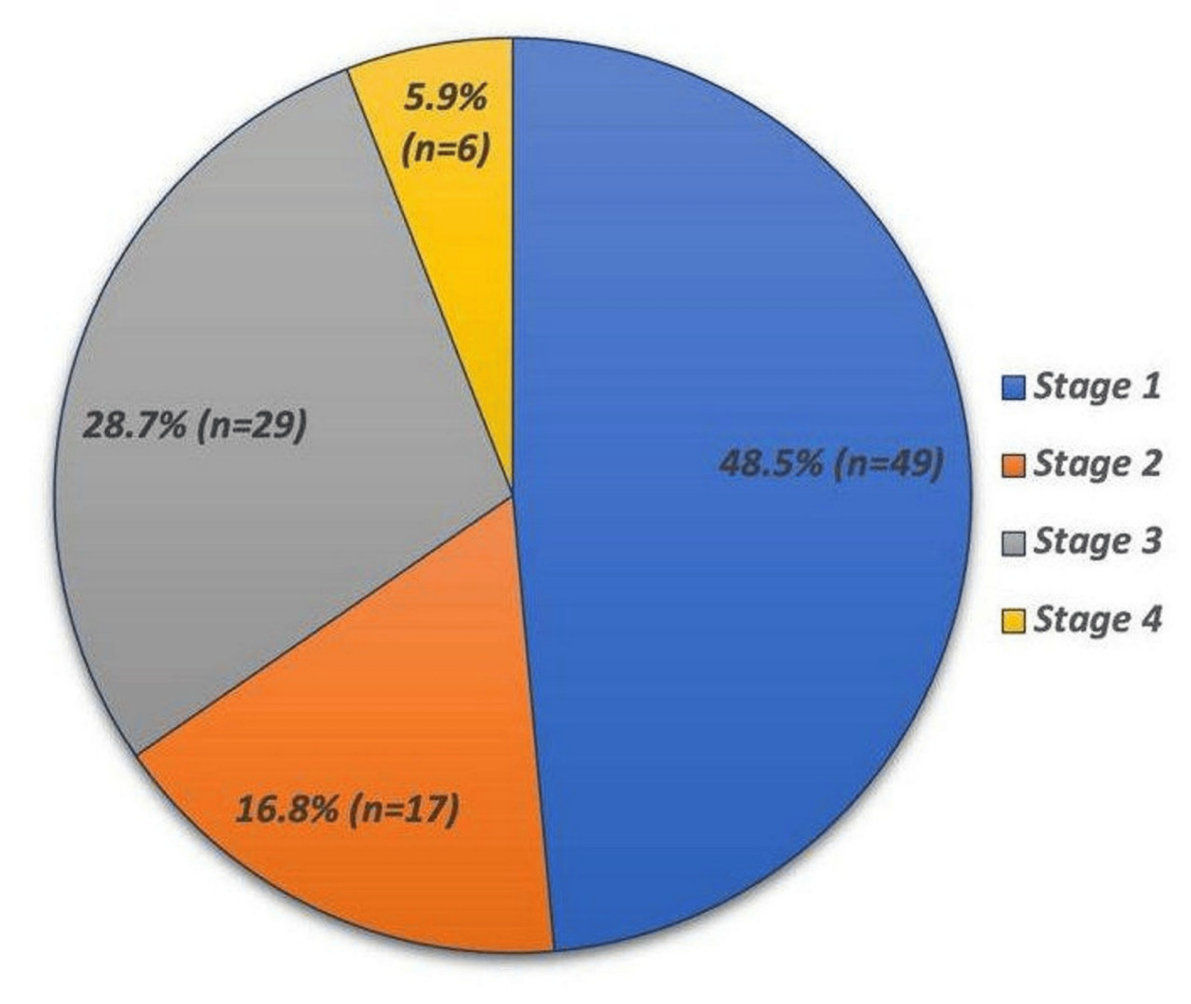
Distribution of stages of epithelial ovarian tumors (n=101)

Figure 2 shows the distribution of epithelial ovarian tumor stages among 101 patients, with an additional 151 patients’ stages not documented in the histopathology report. The majority of cases were stage 1, comprising 48.5% (n=49) of the tumors. This was followed by stage 3, which accounted for 28.7% (n=29) of the cases. Stage 2 was present in 16.8% (n=17) of the participants, while stage 4, the most advanced stage, was the least common at 5.9% (n=6).

Table 3 shows the protective factors, risk factors, and tumor markers associated with ovarian tumors among the patients. Notably, the most significant protective factor was breastfeeding or parity, as reported in 99 patients (39.3%). Hysterectomy was another protective measure, although less common, observed in eight patients (3.2%). Other less frequently reported protective factors, such as oral contraceptives or tubal ligation, were noted in only two cases (0.8%). A significant proportion of the cohort did not report any protective factors (n=143, 56.7%).

**Table 3:** Different protective and risk factors along with tumor markers of ovarian tumors among patients (n=252) CEA: Carcinoembryonic antigen; LDH: Lactate dehydrogenase; CA-125: Cancer antigen 125; CA 19-9: Cancer antigen 19-9; AFP: Alpha-fetoprotein; CA-15.3: Cancer antigen 15.3; HCG: Human chorionic gonadotropin; N/A: Not applicable

Characteristics	Variables	Number of cases (%)
Protective factors	Breastfeeding/parity	99 (39.3%)
	Hysterectomy	8 (3.2%)
	Other	2 (0.8%)
	N/A	143 (56.7%)
Risk factors	Endometriosis	10 (4.0%)
	Family history/genetic predisposition	6 (2.4%)
	History of malignancy	19 (7.5%)
	Leiomyoma	7 (2.8%)
	Multiple risk factors	8 (3.2%)
	Nulliparity	31 (12.3%)
	Other	3 (1.2%)
	N/A	168 (66.6%)
Tumor markers of ovarian cancers	CA-125	93 (36.9%)
	CEA	40 (15.9%)
	CA 19-9	23 (9.1%)
	LDH	22 (8.7%)
	AFP	10 (3.9%)
	CA-15.3	6 (2.4%)
	Estradiol	4 (1.6%)
	HCG	2 (1.9%)

Nulliparity was the most prevalent risk factor, affecting 31 (12.3 %) patients. A history of malignancy was also notable, as reported in 19 patients (7.5%). Other risk factors included endometriosis (n=10, 4.0%), multiple risk factors (n=8, 3.2%), leiomyoma (n=7, 2.8%), and a family history or genetic predisposition (n=6, 2.4%). Other less commonly reported risk factors, such as a history of previous ovarian cysts and polycystic ovary syndrome (PCOS), were observed in three cases (1.2%). The majority of patients did not specify any risk factors (n=168, 66.6%).

Regarding tumor markers, cancer antigen 125 (CA-125) was the most frequently elevated tumor marker and was found in 93 cases (36.9%). This was followed by carcinoembryonic antigen (CEA), observed in 40 patients (15.9%), and cancer antigen 19-9 (CA 19-9) in 23 cases (9.1%). Lactate dehydrogenase (LDH) was present in 22 patients (8.7%) and alpha-fetoprotein (AFP) was reported in 10 patients (3.9%). Less frequent were cancer antigen 15.3 (CA-15.3) and estradiol, noted in six (2.4%) and four (1.6%) patients, respectively, and human chorionic gonadotropin (HCG) was observed in two cases (1.9%).

Table 4 shows the management of ovarian tumors in 252 patients. The predominant treatment was total abdominal hysterectomy with bilateral salpingo-oophorectomy (TAH-BSO) in 92 patients (36.5%). Unilateral ovarian cystectomy was the second most common method used in 51 patients (20.2%). Additionally, 36 patients (14.3%) underwent TAH-BSO combined with chemotherapy, reflecting the use of comprehensive treatment in severe cases. Other methods included unilateral salpingo-oophorectomy (24 cases, 9.5%), bilateral salpingo-oophorectomy, exploratory laparotomy, and chemotherapy, each used in <10 cases. Various lesser-used or adjunctive treatments have also been reported, demonstrating the need for personalized approaches based on specific clinical assessments.

**Table 4:** Treatment approaches for ovarian tumors among patients (n=252) 'Other' includes patients who had other pathologies in the endometrium, cervix, and myometrium with different management combinations: TAH and unilateral salpingo-oophorectomy, TAH-BSO and chemotherapy in addition to radical LN resection and colectomy. TAH: Total abdominal hysterectomy; BSO: Bilateral salpingo-oophorectomy; TAH-BSO: Total abdominal hysterectomy with bilateral salpingo-oophorectomy; LN: Lymph nodes

Treatment Modalities	Number of cases (%)
TAH-BSO	92 (36.5%)
Unilateral ovarian cystectomy	51 (20.2%)
TAH-BSO and chemotherapy	36 (14.3%)
Unilateral salpingo-oophorectomy	24 (9.5%)
BSO	7 (2.8%)
Exploratory laparotomy	7 (2.8%)
Chemotherapy	6 (2.4%)
Bilateral ovarian cystectomy	6 (2.4%)
Hysterectomy	3 (1.2%)
Other	17 (6.7%)

## Discussion

Ovarian cancer, a highly fatal gynecological malignancy, is often diagnosed late because of its asymptomatic early stages [3]. In this study, we analyzed the histomorphological patterns of epithelial ovarian tumors and examined their stages, types, and initial presentations in a diverse female cohort. We explored protective and risk factors to identify preventive measures and risk-reduction strategies. By evaluating tumor markers and treatment protocols, this study highlights the current management effectiveness and potential personalized therapies, contributing to improved detection, treatment, and outcomes in ovarian cancer patients.

Notably, the majority of surface epithelial tumors were serous. This is consistent with global findings that epithelial tumors constitute approximately 90% of ovarian cancers, with serous carcinomas being the most prevalent (Reid et al., 2017) [12]. The frequent identification of serous cystadenomas, a benign variant, suggests a significant detection of tumors at an early stage 1 (48.5%), which is potentially curable. Similarly, Elias et al. (2018) showed that ovarian tumors can be cured if detected early, with up to 90% of patients with stage I ovarian cancer being cured [13].

Within this group, serous epithelial tumors were the most common, comprising 61.1% of the cases. This is in line with global studies that report that serous tumors are the most prevalent (69%) EOCs, particularly in Western countries (Tjokroprawiro et al., 2024) [14]. The breakdown of serous tumors into serous cystadenomas, HGSC, and serous borderline tumors, with HGSC being notably frequent, reflects the trends observed in broader oncology research, indicating that HGSC (50-60%) is the most malignant and most likely to present at an advanced stage (Kim et al., 2018) [15].

Moreover, mucinous epithelial tumors, representing 21.4% of epithelial tumors in this study, with the majority being mucinous cystadenomas, showed a slightly higher range of prevalence compared to the literature averages, which typically range around 6-25% of ovarian tumors (Brown et al., 2014) [16]. This could reflect demographic or environmental differences in the study population. Endometrioid tumors and Brenner tumors, though less common [17], were observed at rates consistent with broader research, underscoring their less frequent occurrence but the potential for a significant impact on patient outcomes.

Notably, there was a high prevalence of stage 1 tumors (48.5%), which is comparable to other studies, such as Gaona-Luviano et al. (2020), where stage 1 tumors accounted for only about 58-64% of cases [18]. The high detection rate of early-stage tumors in the literature could be attributed to improved screening techniques or possibly higher awareness among the population, leading to earlier consultation and diagnosis [19].

Furthermore, the initial presentation of ovarian tumors in our cohort was most commonly abdominal pain, similar to the findings of Dilley et al. (2020), who reported that abdominal pain (41.7%) and mass (25.8%) were the predominant symptoms [20]. However, our study found a higher incidence of incidental findings (23.8%), which highlights the role of routine health check-ups and advanced imaging techniques in the incidental detection of ovarian tumors [21].

In terms of risk factors, nulliparity was the most significant, consistent with the existing literature that suggests nulliparity as a consistent risk factor due to uninterrupted ovulation leading to increased genetic damage over time. Gaitskell et al. (2018) showed that nulliparous women have a 24% higher risk of ovarian cancer than women with one child. The risk increases more than 12-fold compared to that in multiparous women [22]. Interestingly, our study also identified a notable percentage of participants with a family history of malignancy, which supports the theory of shared genetic or environmental risk factors across different cancer types [23].

The protective role of breastfeeding and parity observed in our study corroborates the literature that associates these factors with reduced ovarian cancer risk due to fewer lifetime ovulations (Obeagu et al., 2024) [24]. Less common protective factors, such as hysterectomy and oral contraceptives, have also been recognized in previous studies as methods that decrease the lifetime risk of ovarian cancer (Green et al., 1997) [25].

Tumor marker prevalence, particularly that of CA-125, was extensively noted in our cohort, reflecting its established role as a primary biomarker for ovarian cancer detection and monitoring, as discussed by Scholler et al. (2007) [26]. However, the presence of other markers, such as CEA and CA 19-9, also underscores their potential utility in diagnosing specific tumor types or in cases in which CA-125 is not elevated.

Moreover, the predominance of TAH-BSO in our management approaches is consistent with the standard treatment guidelines for ovarian cancer, which recommend these procedures to limit spread and improve prognosis [27]. The frequent use of chemotherapy, especially in conjunction with surgery, reflects the aggressive nature of ovarian tumors and the necessity for comprehensive treatment strategies to improve survival rates.

This study provides a comprehensive analysis of surface epithelial ovarian tumors in a significant cohort (n=252) from a tertiary care center, offering valuable insights into histopathological patterns, clinical presentations, and management strategies within a regional context. The inclusion of a broad age range enhances its applicability to different patient demographics. The systematic approach to data collection using histopathological evaluations and standardized tumor marker analysis ensures reliability. Moreover, the study highlights region-specific factors such as the protective effects of breastfeeding and parity, which are particularly relevant for public health strategies in similar populations. The use of CA-125 as a marker and detailed staging data contributes to understanding tumor progression and treatment.

Limitations

The retrospective design may introduce selection and recall biases because the data relied on existing medical records. The absence of long-term follow-up limits the ability to assess survival outcomes and the long-term impact of treatment strategies. Additionally, the study's focus on a single center restricts its generalizability to other regions or healthcare settings. Variability in tumor marker expression and incomplete staging data for some patients further constrained the depth of analysis. Future studies with prospective designs and broader sample populations are needed to validate these findings and to address these limitations.

Future direction and implications

Future research on ovarian cancer should use a prospective cohort design for robust causal inferences and include a diverse population for broader applicability. Emphasizing standardized data collection methods, such as digital health records and advanced imaging, will mitigate bias and enhance tumor classification accuracy. Integrating genetic profiling with biomarker analysis can personalize treatment strategies. Long-term studies assessing survival rates and patient quality of life are crucial for evaluating therapeutic effectiveness and refining management strategies for ovarian cancer.

## Conclusions

Our study highlights that surface epithelial tumors, particularly serous epithelial types, are the most prevalent ovarian neoplasms, with abdominal pain commonly serving as the initial symptom. Surgical intervention and chemotherapy are often key to effective management, and factors such as breastfeeding and parity appear to offer significant protective benefits. CA-125 emerged as a frequently utilized tumor marker. This research not only supports established knowledge but also provides valuable insights into regional trends and potential avenues for clinical improvement. Further investigation is needed to understand the reasons behind the high early-stage detection rates and to assess the long-term outcomes of the management strategies employed.

## References

[1] Momenimovahed Z, Tiznobaik A, Taheri S, Salehiniya H (2019). Ovarian cancer in the world: epidemiology and risk factors. Int J Womens Health.

[2] Bray F, Ferlay J, Soerjomataram I, Siegel RL, Torre LA, Jemal A (2018). Global cancer statistics 2018: GLOBOCAN estimates of incidence and mortality worldwide for 36 cancers in 185 countries. CA Cancer J Clin.

[3] Arora N, Talhouk A, McAlpine JN, Law MR, Hanley GE (2018). Long-term mortality among women with epithelial ovarian cancer: a population-based study in British Columbia, Canada. BMC Cancer.

[4] Lowe KA, Chia VM, Taylor A (2013). An international assessment of ovarian cancer incidence and mortality. Gynecol Oncol.

[5] Lheureux S, Gourley C, Vergote I, Oza AM (2019). Epithelial ovarian cancer. Lancet.

[6] Fitch M, Deane K, Howell D, Gray RE (2002). Women's experiences with ovarian cancer: reflections on being diagnosed. Can Oncol Nurs J.

[7] Matulonis UA, Sood AK, Fallowfield L, Howitt BE, Sehouli J, Karlan BY (2016). Ovarian cancer. Nat Rev Dis Primers.

[8] Gilks CB, Prat J (2009). Ovarian carcinoma pathology and genetics: recent advances. Hum Pathol.

[9] Jadhav PN, Jadhav R (2020). Clinicopathological study of surface epithelial ovarian tumors - a tertiary care center study. Paripex Indian J Res.

[10] Kim SI, Lim MC, Lim J, Won YJ, Seo SS, Kang S, Park SY (2016). Incidence of epithelial ovarian cancer according to histologic subtypes in Korea, 1999 to 2012. J Gynecol Oncol.

[11] Sait KH, Alam MZ, Haque A, Sait HK, Sait MK, Anfinan NM (2022). Survival and prognostic factors in women treated for epithelial ovarian cancer in western region of Saudi Arabia. Saudi Med J.

[12] Reid BM, Permuth JB, Sellers TA (2017). Epidemiology of ovarian cancer: a review. Cancer Biol Med.

[13] Elias KM, Guo J, Bast RC Jr (2018). Early detection of ovarian cancer. Hematol Oncol Clin North Am.

[14] Tjokroprawiro BA, Novitasari K, Ulhaq RA, Sulistya HA (2024). Clinicopathological analysis of giant ovarian tumors. Eur J Obstet Gynecol Reprod Biol X.

[15] Kim J, Park EY, Kim O, Schilder JM, Coffey DM, Cho CH, Bast RC Jr (2018). Cell origins of high-grade serous ovarian cancer. Cancers (Basel).

[16] Brown J, Frumovitz M (2014). Mucinous tumors of the ovary: current thoughts on diagnosis and management. Curr Oncol Rep.

[17] Costeira FS, Félix A, Cunha TM (2022). Brenner tumors. Br J Radiol.

[18] Gaona-Luviano P, Medina-Gaona LA, Magaña-Pérez K (2020). Epidemiology of ovarian cancer. Chin Clin Oncol.

[19] Crosby D, Bhatia S, Brindle KM (2022). Early detection of cancer. Science.

[20] Dilley J, Burnell M, Gentry-Maharaj A (2020). Ovarian cancer symptoms, routes to diagnosis and survival - Population cohort study in the 'no screen' arm of the UK Collaborative Trial of Ovarian Cancer Screening (UKCTOCS). Gynecol Oncol.

[21] Liberto JM, Chen SY, Shih IM, Wang TH, Wang TL, Pisanic TR 2nd (2022). Current and emerging methods for ovarian cancer screening and diagnostics: a comprehensive review. Cancers (Basel).

[22] Gaitskell K, Green J, Pirie K, Barnes I, Hermon C, Reeves GK, Beral V (2018). Histological subtypes of ovarian cancer associated with parity and breastfeeding in the prospective million women study. Int J Cancer.

[23] (2002). The links between environmental factors, genetics, and the development of cancer. Cancer and the Environment: Gene-Enviroment Interaction.

[24] Obeagu EI, Obeagu GU (2024). Breastfeeding's protective role in alleviating breast cancer burden: a comprehensive review. Ann Med Surg (Lond).

[25] Green A, Purdie D, Bain C, Siskind V, Russell P, Quinn M, Ward B (1997). Tubal sterilisation, hysterectomy and decreased risk of ovarian cancer. Survey of women's health study group. Int J Cancer.

[26] Scholler N, Urban N (2007). CA125 in ovarian cancer. Biomark Med.

[27] Kim I (2019). Intraoperative consultation for ovarian tumors. Yeungnam Univ J Med.

